# Diagnosis of congenital CMV infection via DBS samples testing and neonatal hearing screening: an observational study in Italy

**DOI:** 10.1186/s12879-019-4296-5

**Published:** 2019-07-22

**Authors:** Laura Pellegrinelli, Cristina Galli, Valeria Primache, Mirko Alde’, Enrico Fagnani, Federica Di Berardino, Diego Zanetti, Elena Pariani, Umberto Ambrosetti, Sandro Binda

**Affiliations:** 1 Dipartimento di Scienze Biomediche per la Salute, Università degli Studi di Milano, Via Carlo Pascal, 36, 20133 Milan, Italy; 2 Dipartimento di Scienze Cliniche e di Comunità, Università degli Studi di Milano, Milan, Italy; 3 U.O.S.D di Audiologia, Fondazione I.R.C.C.S. Ca’ Granda Ospedale Maggiore Policlinico di Milano, Milan, Italy

**Keywords:** Congenital Cytomegalovirus, Neonatal hearing screening program, Hearing loss, Dried blood spot

## Abstract

**Background:**

Congenital Cytomegalovirus (cCMV) is the most common cause of non-genetic hearing loss in childhood. A newborn hearing screening program (NHSP) is currently running in Italy, but no universal cCMV nor statewide hearing-targeted CMV screening programs have been implemented yet.

This observational monocentric study was aimed at estimating the rate of cCMV infections identified by CMV-DNA analysis on Dried Blood Spots (DBS) samples in deaf children identified via NHSP in Northern Italy in the period spanning from 2014 to 2018.

**Methods:**

Children with a confirmed diagnosis of deafness and investigated for CMV-DNA by nucleic acid extraction and in-house polymerase-chain reaction (PCR) on stored newborns screening cards (DBS-test) were included in this study. Deafness was defined by a hearing threshold ≥20 decibel (dB HL) by Auditory Brainstem Responses (ABR); all investigated DBS samples were collected within 3 days of life.

**Results:**

Overall, 82 children were included (median age: 3.4 months; lower-upper quartiles: 2–5.3 months; males: 60.9%). Most of them (70.7%) presented bilateral hearing loss with a symmetrical pattern in 79.3% of the cases. ABR thresholds were ≥ 70 dB HL (severe/profound deafness) in 46.5% of children. Among all tested children, 6.1% resulted positive for cCMV. The rate of severe/profound deafness was statistically higher in children with cCMV infection.

**Conclusions:**

The addition of DBS-test to the NHSP allowed the identification, in their first months of life, of a cCMV infection in 6.1% of children who had failed NHS. The introduction of a targeted CMV screening strategy could help clinicians in the differential diagnosis and in the babies’ management. DBS samples can be considered a “universal newborns biobank”: their storage site and duration should be the subject of political decision-making.

## Background

Cytomegalovirus is the leading cause of congenital infection (cCMV) affecting up to 0.8% of newborns in high income countries [1]. A wide range of diseases, diagnosed either at birth or in childhood, has been correlated to cCMV infection [1]; sensorineural hearing loss is the most frequent permanent damage, occurring in 5–40% of infected children, either symptomatic or not at birth; as a matter of fact, cCMV is the most common cause of non-genetic deafness in childhood [2]. An early identification of infected children – ideally through a universal neonatal screening - might allow a prompt treatment, aimed at limiting the hearing loss progression and possibly preventing the onset of cCMV-linked damages to the central nervous system in both symptomatic and asymptomatic babies [3, 4].

According to the recent literature, the virological investigations for cCMV in infants failing their newborn hearing screening program (NHSP) - which is performed in many countries- could overcome the absence of an universal cCMV screening [3–6].

The diagnosis of cCMV infection can be achieved by the examination of salivary/urinary samples collected within the first 2–3 weeks of age as the results of NHSP [3–6]; after this early period of life, it is possible to distinguish between congenital and acquired CMV infection just by examining the Dried Blood Spot (DBS) samples on the ‘Guthrie’ cards that are universally collected within three days from the birth. These Guthrie cards are obtained in all newborns for the screening of metabolic and genetic disorders and stored at the Regional neonatal screening centre. Currently, a nationwide NHSP is in place, but neither a universal cCMV nor a nationwide hearing-targeted CMV screening programs are ongoing in Italy.

This observational single-centre study was aimed at estimating the rate of cCMV infection in children with hearing loss identified via NHSP who were referred to a Pediatric Audiology department in Lombardy (Northern Italy) between 2014 and 2018.

cCMV infection was diagnosed through the virological examination of DBS retrieved from the Regional neonatal screening centre.

## Methods

### Study design

This observational monocentric study was conducted in Milan (Italy) between January 1^st^ 2014 and December 31^th^ 2018.

All children belonging to the 2014–2018 birth cohort failing NHSP with transient-evoked optoacoustic emissions (TEOAE) in one or both ears and that also underwent Automated Auditory Brainstem Responses (A-ABR) were eligible in the study. Children who showed abnormal A-ABR recordings referred to the Audiology Department of the “Fondazione IRCCS Ca’ Granda, Ospedale Maggiore Policlinico” and with an informed consent for retrieving and testing of DBS samples for cCMV diagnosis were included in the study.

Data of included children encompassed gender, age, age at diagnosis of hearing loss; information on hearing loss severity, laterality and occurrence of unilateral hearing loss were also collected at the enrollment.

The study population was categorized according to the result of the ABR assessment:

i) with mild/moderate (20–70 dB HL) vs. severe/profound (> 70 dB HL) hearing loss according to ABR Wave threshold;
ii) affected by unilateral vs. bilateral hearing loss;
iii) presented with bilateral symmetric or asymmetric hearing loss pattern. When hearing loss was asymmetric, the worse ear was considered in the hearing loss level allocation.

#### Ethics approval and consent to participate

Written informed consent was obtained from the legal guardian of the children. The study was performed according to the guidelines of the Institutional Review Board on the use of biological specimens for scientific purposes in keeping with Italian law (art.13 D. Lgs 196/2003). Since this was a retrospective study on the results already available with us, it was not put up for any clearance to an ethical committee. The data were handled anonymously. The study accomplished the Helsinki Declaration of 1975, rev. 2000.

### ABR assessment

Clinical ABR determination of hearing threshold was carried out at the Audiology Department at the “Fondazione IRCCS Ca’ Granda, Ospedale Maggiore Policlinico di Milano”, a third level referral audiological centre in Milan. In brief, babies were tested under spontaneous sleep conditions in a soundproof and Faradized room; Ag-AgCl electrodes were stuck in a conventional A1-A2-Cz-Fpz configuration and connected to the Navigator Pro (Natus Bio-logic, California, USA) recording apparatus.

The stimuli were biphasic clicks delivered monaurally via a TDH-39 (Smart Diagnostic Devices, Minnesota, USA) transducer at a repetition rate of 11pps with 10 dB HL steps of decreasing in intensity until the wave V became indistinguishable from the baseline. Responses to 2000 clicks were averaged. For each recording condition the trials were repeated at least twice.

When a diagnosis of deafness was confirmed for a child, an informed consent for retrieving and testing of DBS samples for cCMV was obtained from parents.

### CMV-DBS test

Between January 1^st^ 2014 and December 31^th^ 2018, DBS samples collected from children with a diagnosis of deafness were retrieved from the regional neonatal screening centre and tested for CMV at the Virological Laboratories of the Department of Biomedical Sciences for Health, University of Milan.

DBS samples were processed by two separate in-house steps consisting of DNA extraction from the DBS samples by a heat-extraction-method, followed by amplification of a fragment of the CMV genome using a nested-PCR. Briefly, 3 × 3 mm punches (1 punch/tube; area of single punch: 7.1 mm^2^; overall area of punches: 21.3 mm^2^) of DBS were collected from each ‘Guthrie’ card. The punches were initially incubated in 35 μL of Minimum Essential Medium plus Earle’s solution (EuroClone SpA, Milano, Italy) overnight at 4 °C, followed by 60 min at 56 °C and then heated for 7 min at 100 °C. After 3 min of centrifugation (10,000 rpm) the supernatants were frozen for 1 h at −80 °C. An house-keeping gene was amplified with a PCR designed to recognise human beta-2 microglobulin, as previously described [7], followed by a nested-PCR designed to amplify the CMV UL55 gene [8]. The first round-PCR conditions consisted of 1 cycle of 2 min at 95 °C, followed by 35 cycles of 30 s at 95 °C, 30 s at 58 °C, 30 s at 72 °C and a final cycle of 5 min at 72 °C. The second round-PCR conditions consisted of 1 cycle of 15 min at 95 °C, followed by 30 cycles of 30 s at 95 °C, 30 s at 50 °C, 30 s at 72 °C and a final cycle of 5 min at 72 °C [8]. The lower limit of detection of the CMV-DBS test is 400 CMV-DNA copies/mL.

### Data analysis

The statistical analysis was performed using the Open Source Epidemiologic Statistics for Public Health (OpenEpi, Georgia, USA) version 3.03. The frequencies were expressed as a crude proportion with a corresponding 95% confidential interval (CI), assuming a normal distribution. Proportions between groups were compared using the Mid-P exact test based on binomial distribution. Lower (Q1) and upper (Q3) quartiles were computed. The risk of infection was expressed as the number of individuals with a laboratory-confirmed cCMV infection out of the total number of children with hearing loss and a specific characteristic. Odd Ratio (OR) and exact CI were computed. The two-sample *t*-test and the Fisher’s exact test were used for continuous and categorical variables, respectively. *P* values of <.05 were considered to be statistically significant (two-tailed test).

## Results

During the 4 years recruitment period, 89 children failed NHSP. For 82 of them (92.1%) the DBS samples were available and included in the study. For 7 children DBS samples were not retrieved because stored out of our regional screening center or the informed consent was not obtained thus were excluded from the data analysis.

For children included in the study, the median age for ABR diagnosis after NHSP was 3.4 months (Q1: 2 months - Q3: 5.3 months). Most hearing loss diagnoses (66/82; 80.5%) were achieved before the age of 6 months and more than half of them (37/66; 56%) were obtained before the age of 3 months. No differences in distribution by gender were observed: 50/82 (60.9%) were males (*p*-value =0.08).

Seventy-seven percent of children (58/82; p-value <.0001) presented with bilateral hearing loss that showed a symmetrical pattern in 79.3% (46/58; 95% CI: 67.2–87.7%; *p*-value <.0001) of cases. ABR threshold resulted ≥70 dB HL (severe/profound deafness) in 46.5% of the children (38/82; 95% CI: 35.9–57.0; p-value =0.90). Twenty-eight out of 82 children (34.1%; 95% CI: 24.8–44.9%), required hearing aids.

The timing of the DBS samples’s retrieval and examination was marked by the timing of hearing diagnoses by ABR. According to the virological investigation, 6.1% (5/82; 95% CI: 2.6–13.5%) of DBS samples tested positive for cCMV. The rate of children with severe/profound deafness was significantly higher in those with cCMV infection than in the remaining others: 100% vs 42.9% respectively (p-value =0.018).

No difference between unilateral and bilateral hearing loss (OR = 0.6; 95% CI: 0.08–5.2) and between symmetrical vs. asymmetrical hearing loss (OR = 0.6; 95% CI: 0.07–5.1) was evidenced comparing cCMV-positive and cCMV-negative children.

## Discussion

cCMV infection is a very serious public health issue, either for its significant prevalence - up to 1% of all newborns - and for the severe and permanent sensorial and neurological impairments it can cause [1]; among these, hearing loss has the highest impact on the children’s health, endangering their neurological, cognitive and behavioural development [2].

Hearing loss due to cCMV infection can be already detected at birth, but a diagnosis is commonly established later on, usually during the first years of life, owing to the often delayed and progressive manifestation of the neurological damage [2]. The best preventive strategy would be the implementation of a universal neonatal cCMV screening, being able to identify the totality of cCMV-positive children. It would also enable an earlier identification and correction of the main cause of hearing loss with post-natal onset [5]. If, for any reason, a neonatal cCMV screening is not feasible on a large scale, at least a hearing-targeted cCMV screening should be set up: it would allow the early identification of children with cCMV-related hearing loss, although it would miss delayed hearing losses or other cCMV-related disease [3, 4, 9]. The following benefits from hearing-targeted CMV screening can be underlined; i) it would provide an accurate assessment of the aetiology of childhood deafness, helping physicians in children’s management and follow-up; ii) it would allow an adequate counselling and support to parents; iii) it would help predicting whether another child might be born with hearing-linked damages and, finally, iv) it could alert clinicians on the possible neurological and behavioural complications. In addition, hearing-targeted cCMV screening has demonstrated to be cost-effective for the public health perspective and feasible for health-care facilities in highly resourced settings [9, 10].

At present, no universal- or hearing-targeted cCMV screening is operating in Italy; Tuscany is the only region where all the newborns who failed the first level screening with TEOAE are tested for urinary CMV DNA within the 15th day after birth [11].

In our observational single-institution study, hearing loss has been diagnosed via NHSP, whereas the ascertainment of the cCMV nature of the damage was possible through the examination of DBS samples collected at birth, whose usefulness for cCMV diagnosis has been widely demonstrated [10, 12–15]. In agreement with Boudewyns et al. [12], the investigated children were on average 3.4 months old, far beyond the age (2–3 weeks) at which a congenital infection can be diagnosed through the classical analysis of urinary/salivary samples, as it is planned in a standard cCMV hearing targeted screening.

DBS are the only samples routinely collected at birth and stored, for a short or extended time depending upon each country policies; thus, they represent a ‘universal newborns biobank’ that offers the best chance to get a retrospective cCMV diagnosis.

Even though the sensitivity of DBS testing can be highly variable since it is strongly affected by the blood component release from ‘Guthrie’ Card and by the nucleic acid extraction methodology applied for [12–15], the outcomes of the present study are in agreement with the literature: cCMV infection was identified in 6.1% of children, similarly to other reports in which the rate of cCMV in children failing NHSP ranged between 5 and 8% [3, 4, 12]. The workflow of an hearing-targeted cCMV screenings can also been realized by CMV PCR testing on saliva and/or urine [3]; however, in this setting, we absolutely needed to have the results of audiological examination (TEOAE and ABR) before children were 21 days old days and prior the hospital discharge of the children in order to collect saliva and urine that - unlike in the case of DBS - are not routinely sampling at birth [3].

As far as the correlation of cCMV infection to severity and laterality of hearing loss is concerned [13, 16], in our series the percentage of severe/profound deafness was statistically higher (*p* = 0.018) in children with cCMV infection (100%) than in the rest of the group (42.9%), making the risk to develop a severe/profound deafness higher in children with cCMV infection. This implies that likelihood of developing severe/profound deafness is much higher in the event of cCMV, confirming the trend observed by other researches [13, 16].

A review from Grosse et al. [2] showed that the bilaterality of deafness caused by cCMV is unpredictable, ranging from 25 to 80%. In our study, bilateral hearing loss developed in 71% of cCMV-positive children; no statistical difference between the occurrence of unilateral vs. bilateral hearing loss was evidenced.

Regardless of hearing loss aetiology, children language outcomes depend on time of deafness’ recognition and can be improved with a diagnosis within 6 months of age [3, 4, 6, 17].

## Conclusions

In conclusion, using the DBS-test based on NHSP allowed to identify cCMV infection in 6.1% of children failing NHSP in their first months of life, and to recognize that the risk to develop severe/profound deafness was higher in the event of cCMV. We are convinced that it is time to move toward a hearing-targeted CMV screening strategy, that would provide undoubted benefits for patients and clinicians [6, 16]. Moreover, hearing-targeted cCMV screening yields relevant implications from the public health perspectives. Besides the economic savings expected with the reduction of expenses for social support when CMV damages are prevented or treated earlier [9], the early recognition of cCMV can drive further public health and social initiatives; all these, can improve the community outreach and the health workers in-depth knowledge about cCMV infection and its prevention.

Finally, DBS samples can be considered a “universal newborns biobank”: the site and the duration of their storage should be addressed in political decision-making. The alternative of leaving the DBS samples to parents could overcome the fear of loss of privacy and the costs for a correct samples storage.

## Data Availability

We declare that the raw data analysed in this study can be available upon request from Laura Pellegrinelli (corresponding author).

## Abbreviations

A-ABR: Automated Auditory Brainstem Responses
cCMV: congenital Cytomegalovirus infection
CI: Confidential interval
dB HL: decibel in hearing level
DBS: Dried Blood Spot
NHSP: Newborns Hearing Screening Program
OR: Odd Ratio
PCR: Polymerase chain reaction
Q1: Lower quartiles
Q3: Upper quartiles
TEOAE: Transient-evoked optoacoustic emissions
